# Interfacial Insights into the Polarization Protocol: Toward Reducing Corrosion and Improving the Cycle Life of Electrochemical Capacitors

**DOI:** 10.1021/acsami.4c00767

**Published:** 2024-05-18

**Authors:** Jarosław Wojciechowski, Katarzyna Szwabińska, Krzysztof Fic, Grzegorz Lota

**Affiliations:** † Institute of Chemistry and Technical Electrochemistry, Poznan University of Technology, Berdychowo 4, Poznan 60-965, Poland; ‡ Faculty of Chemistry, Department of Inorganic and Analytical Chemistry, Electrochemistry@Soft Interfaces Team, University of Lodz, Tamka 12, Lodz 91-403, Poland; § Łukasiewicz Research Network − Institute of Non-Ferrous Metals Division in Poznan, Central Laboratory of Batteries and Cells, Forteczna 12, Poznan 61-362, Poland

**Keywords:** corrosion, current collectors, energy storage devices, stainless steel, supercapacitors

## Abstract

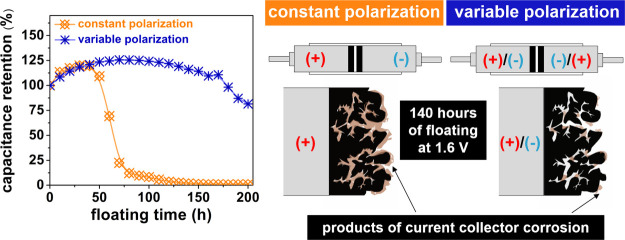

The number of scientific publications on the impact of corrosion on current collectors on the working parameters of electrochemical capacitors is very limited. The aim of current research is to search for new, environmentally friendly chemical power sources and energy storage devices and to improve existing ones. Therefore, this article presents a simple and effective way to improve the life of a symmetric electrochemical capacitor by changing the direction of electrode polarization, which in turn inhibits the corrosion of the current collector. This slows the degradation of current collectors of positive electrode over long durations. However, activated carbon electrode corrosion also occurs. Experiments on capacitors with stainless steel and gold current collectors indicate that the lifespan of the latter is much longer than that of the former. Therefore, current collector corrosion has a distinct and detrimental impact on electrochemical capacitor operation. Moreover, the research results indicate that carbon corrosion results from current collector corrosive damage.

## 1 Introduction

Electrochemical capacitors (ECs) are devices capable of storing charge directly in an electric double layer at electrode/electrolyte interfaces or indirectly when charge transfer process across electrode/electrolyte interface is involved.^1−32^ Unlike batteries, ECs can be fully charged and discharged in a few seconds. Therefore, they are characterized by high power but low energy densities.^1−5^ Furthermore, due to the use of carbon materials with high specific surface areas (up to 2500 m^2^/g), the amount of accumulated charge in ECs is several times higher compared to conventional electric and electrolytic capacitors.^33−36^ For the above reasons, electrochemical capacitors and batteries are critical parts of power systems that are used in industry and everyday life.

Electrochemical capacitors consist of two porous electrodes, a separator, and current collectors. The active electrode material and the separator are soaked in an electrolyte solution (aqueous, organic, or ionic liquid).^37^ The kind of electrolyte used determines several working parameters in the entire system. Devices with aqueous electrolytes operate at a voltage lower than those with an organic electrolyte due to the electrochemical decomposition of water (1.23 V). This makes the energy density value of these ECs markedly lower. The only exceptions are aqueous solutions of alkali metal salts, especially those based on sulfates. In this case, the value of the EC operating voltage reaches ∼1.6 or even 2.0 V.^38^ The advantages of using aqueous electrolytes are the low cost, higher conductivity, and thus higher power density value of such systems and their safe utilization properties, i.e., nonflammability and low toxicity.

All electrical energy storage devices, including ECs, deteriorate after some time with use, i.e., a gradual decline in their working parameters is observed. This is due to the destruction and degradation of the carbon electrode materials, current collectors, and separators, as well as the decomposition of the electrolyte solution.^30−32,39−42^ Undoubtedly, the main reason is the corrosion of the collectors, i.e., the stainless steel or metal elements responsible for conducting electric charge. This phenomenon is more pronounced if the electrolyte is aqueous. The corrosion process of current collectors in battery systems, especially in lead-acid and lithium-ion systems, as well as the influence of this process on the working parameters of these devices, has been described in depth in the last 20 years.^43−46^ Unfortunately, in the case of electrochemical capacitors, the number of publications is still moderate.

In this paper, we present a simple and practical way to extend the life of an electrochemical capacitor system by inhibiting corrosion of the current collectors. Piwek et al.^47^ noted that carbon corrosion was the cause of the destruction of an electrochemical capacitor. We extend this research and elaborate the reasons for the destruction of capacitor systems operating in aqueous electrolyte solutions. Our intention is to present the corrosion mechanism of current collectors that underlies the degradation of the entire capacitor system.

Recognizing the sources of carbon corrosion, and thus the degradation of the device, will allow for a broader understanding of the lifespan of electrochemical capacitors and more efficient measures to prevent degradation. The results obtained from electrochemical tests clearly indicate that the lifetime of electrochemical capacitors is extended when they are operated in variable electrode polarization mode. Therefore, we postulate that the corrosion of current collectors is the primary factor leading to the destruction of capacitors.

## 2 Experimental Section

### 2.1 General Information

All electrochemical measurements were performed at ambient temperature using an electrochemical workstation potentiostat/galvanostat VMP 3 (Biologic, France) with an impedance module. All chemicals were purchased from Sigma–Aldrich.

### 2.2 Assembly of Electrochemical Capacitors

Symmetric electrochemical capacitors were constructed as Swagelok cell systems intended for two- and three-electrode electrochemical tests. A description of such a system can be found elsewhere.^30−32,40^ Carbon electrodes were prepared in the form of pellets (12 mm in diameter) with a mass of ∼10 mg. The electrodes were composed of 85 wt % activated carbon (Kuraray YP 80F), 10 wt % binder (Teflon) and 5 wt % carbon black (acetylene black). The separator and carbon electrode materials were soaked with 1 M Na_2_SO_4_ solution. Cylinders made of 316 L stainless steel served as current collectors. The nominal composition of stainless steel is presented elsewhere.^30−32,40^ Furthermore, the following surface treatment of current collectors was carried out: degreasing in acetone (15 min) and in hot (85 °C) 10% KOH solution (15 min); rinsing with distilled water (15 min); drying in an oven at 80 °C (30 min); and aging (conditioning) in air at 25 °C (24 h).

### 2.3 Testing of Electrochemical Capacitors

#### 2.3.1 Stage I of Electrochemical Tests

Two identical electrochemical capacitors were constructed and subjected to the following electrochemical tests: electrochemical impedance spectroscopy (EIS), cyclic voltammetry (CV), galvanostatic charging/discharging (GCD), open circuit voltage (OCV) measurement, and floating (potentiostatic technique). The capacitors were charged to a voltage of 1.6 V. First, the two-electrode tests were performed in the following order: EIS (amplitude ±10 mV, frequency 100 kHz–10 mHz, 0.000 V vs OCV); CV (1–100 mV s^–1^); EIS and GCD (100–1000 mA g^–1^). This was the first stage of the research, which was carried out once.

#### 2.3.2 Stage II of Electrochemical Tests

In the second stage, an accelerated aging procedure was applied. The purpose of applying this procedure was to estimate the lifetime of both capacitors and to achieve a certain state of deterioration. The systems were charged by the GCD technique and kept charged for 5 h (1.6 V) (so-called floating step). Then, the capacitors were subjected to GCD and CV tests. The open-circuit voltage was then measured for a period of 1 h. Finally, the EIS test (at 0.000 V vs OCV) was performed. The procedure was repeated. In total, the capacitor was charged for a period of 10 h in one cycle. Stage II was repeated over 20 cycles, i.e., the capacitors were fully charged for 200 h. Each cycle was repeated on the following day at the same time. The capacitors were in open circuit conditions until the beginning of each cycle, i.e., for approximately 9 h. This was the time between the end of the last cycle and the start of a new cycle. The terminals of one of the systems remained unchanged throughout the entire period of the tests (stage II), i.e., the positive and negative electrodes were kept constant, and such a capacitor was marked as a CP (constant polarization). The second system was characterized by a change in electrode polarization, i.e., the capacitor marked as VP (variable polarization) was charged alternately to a voltage of 1.6 V in one cycle and to −1.6 V in the following cycle. In this case, each electrode was polarized anodically (10 cycles) and cathodically (10 cycles) during stage II.

To confirm the hypotheses regarding the collector corrosion influence, stage II electrochemical measurements were repeated also for the same electrochemical capacitor cell systems built with gold current collectors.

#### 2.3.3 Stage III of Electrochemical Tests

Stage III included three-electrode measurements (EIS at 0.000 V vs OCP), which were performed to assess the impact of the long-term aging procedure (stage II) on the degradation of individual electrodes in both systems exactly 1 week after the end of stage II. This allowed us to estimate the influence of the application of the variable polarization mode on the working parameters of the electrochemical capacitor.

#### 2.3.4 Stage IV of Electrochemical Tests

Stage IV involved three-electrode electrochemical tests (EIS at 0.000 V vs OCP) in four different electrochemical capacitor systems. These systems were composed of two different components from all electrochemical capacitors tested in all previous stages. These components were the carbon materials and current collectors. The four different systems were constructed as follows: (i) fresh carbon materials and the current collectors of the CP capacitor utilized in the previous stages (CP1); (ii) the carbon materials of the CP capacitor (previous stages) and fresh current collectors (CP2). Analogous test systems were constructed based on the materials of the VP capacitor: (iii) VP1 and (iv) VP2. All fresh materials were prepared as mentioned above. Stage IV measurements were made exactly 1 week after the end of stage III.

### 2.4 Additional Electrochemical Tests

The surfaces of current collectors in Swagelok systems are difficult to characterize by means of morphology analysis and physicochemical techniques. Therefore, to accurately reproduce the conditions affecting their surface, the electrochemical regime of 316 L stainless steel discs under stage II conditions, presented above, was carried out. Two-electrode systems were constructed in plexiglass cells. Two discs made of 316 L stainless steel with a diameter of approximately 28 mm were appropriately prepared, like the previous current collectors, and were placed opposite to each other at a distance of 10 cm. The space between them was filled with 1 M Na_2_SO_4_ electrolyte solution. It is very important to keep in mind that the above tests for steel discs in plexiglass cells do not fully reflect the phenomena occurring on the surface of the current collectors in the Swagelok systems, but certain conditions could be simulated. Nevertheless, they revealed the differences in the rate and mechanism of current collector corrosion in two different electrochemical capacitors.

Using the above approach, 316 L stainless steel discs with corrosion-affected surfaces were obtained. The surface was disturbed as a result of the use of constant and variable polarization in plexiglass cells. The elements obtained in this way were subjected to three-electrode electrochemical tests, again in plexiglass cell vessels. The working electrodes were stainless steel discs, while platinum and mercury sulfate electrodes were used as counter electrodes and reference electrodes, respectively. Analysis was carried out in a 1 M Na_2_SO_4_ electrolyte solution using electrochemical impedance spectroscopy (at 0.000 V vs OCP) and cyclic potentiodynamic polarization (CPP) techniques.

### 2.5 Nonelectrochemical Characterization

Additionally, some elements were subjected to surface morphological and physicochemical analyses immediately after the electrochemical tests. Nonelectrochemical characteristics were determined for carbon electrode materials (ECs cells), separators (ECs cells), and 316 L stainless steel disc surfaces after aging tests (according to stage II) in plexiglass-type cells. Surface images were taken with three different types of microscopes, i.e., a Keyence microscope, a scanning electron microscope (SEM), and an atomic force microscope (AFM). Using AFM, surface roughness and depth of scratches on 316 L stainless steel surfaces, which were made with a steel lancet, were analyzed. Additionally, energy-dispersive X-ray spectroscopy (EDS) and X-ray photoelectron spectroscopy (XPS) were performed to confirm the presence of possible corrosion products on the surfaces of the stainless steel and to characterize them, the carbon material and the separator. To analyze surface morphology and carry out physicochemical tests, the following apparatus was used: a Keyence VHX-7000 digital microscope; an Agilent 5500 atomic force microscope (AFM); an FEI Quanta 250 FEG scanning electron microscope equipped with an energy-dispersive spectroscopy detector (EDS); and a multichamber UHV analytical system (Specs) (XPS analysis).

## 3 Results and Discussion

**Figure 1:**
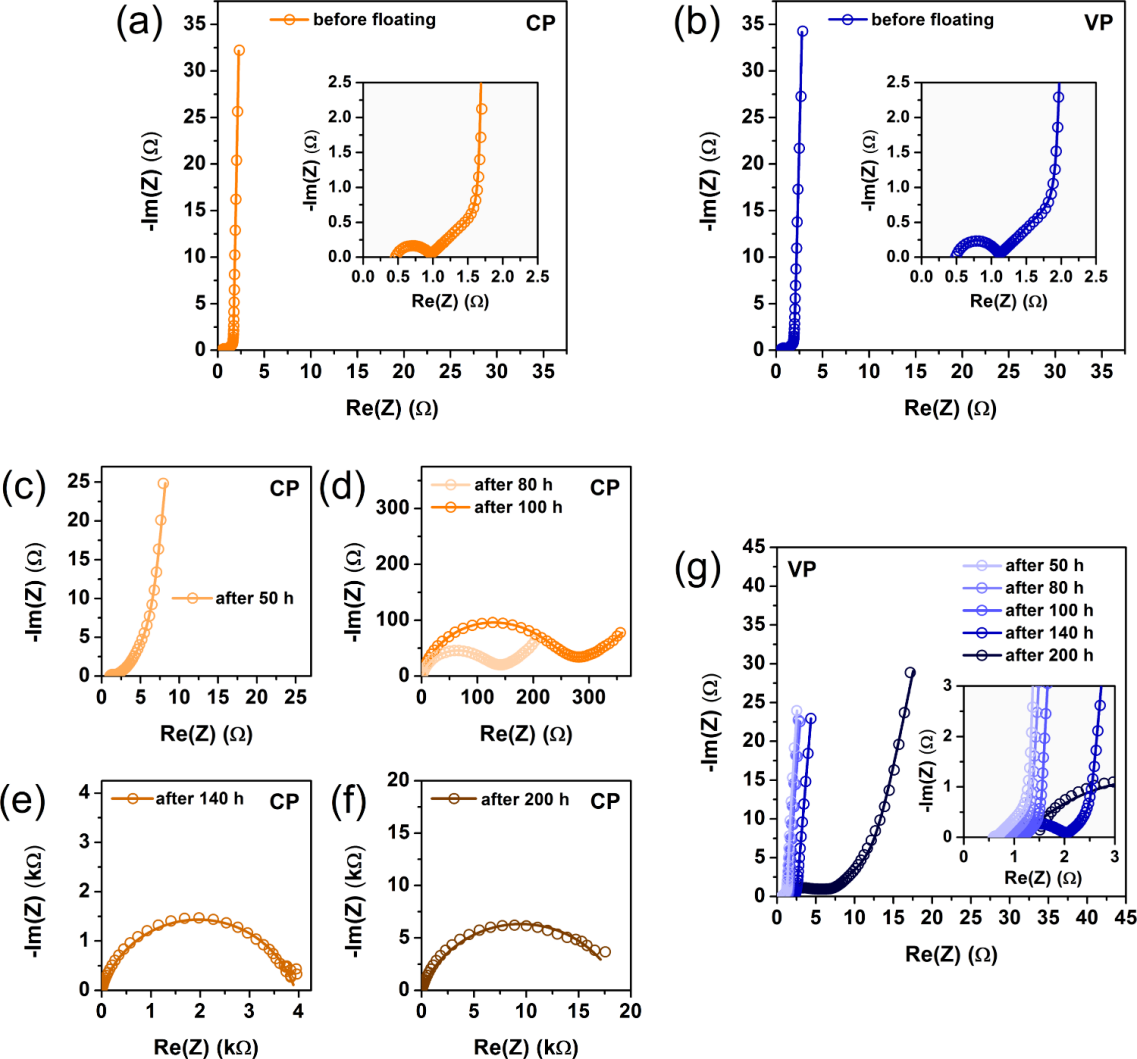
Nyquist plots (recorded in stage I (a, b) and II (c–g)) for capacitors operating in (a, c–f) constant polarization mode and (b, g) variable polarization mode. Experimental points are given as symbols, whereas fitted curves are represented by solid lines.

Figure 1a,b shows the results of the initial EIS tests performed in the first stage of research for capacitors CP and VP.

As expected, the Nyquist curves are initially the same for both cells. The difference in EIS response between capacitors CP and VP becomes noticeable in the second stage of the investigation, i.e., when they are subjected to floating at constant and variable polarization modes, respectively.

As shown in Figure 1c–f, in the second stage, the constant polarization (CP) capacitor is destroyed, i.e., the nature of the Nyquist curves changes. The values of the imaginary and real parts of the impedance are very large in this case. The CP system is completely damaged in contrast to the capacitor with variable polarization (VP), for which the EIS spectra are presented in Figure 1g. The change in the nature of the Nyquist curves can be visualized on the basis of changes in the electrical equivalent circuit (EEC), which was fitted to the obtained EIS results. Figure S1 (Supporting Information (SI) material) presents three different EECs that were used to fit the EIS data. Table 1 presents the values of all individual components of the EECs, matched to the results of the EIS tests for the CP capacitor in stages I and II. Analogous data for the VP capacitor are given in Table 2. Notably, the nature of the Nyquist curves (and corresponding EEC) changes over the duration of the experiment only in the case of capacitor CP. The EEC presented in Figure S1a was fitted to the results of the stage I tests (both CP and VP). This circuit includes elements such as Q, R and M. Their definition and meaning have been presented in previous studies.^30,48−50^ However, short explanation is also given in the Supporting Information (SI) material.

**Table 1:** Values of Individual Components of Electrical Equivalent Circuits Presented in Figure S1 Matched the EIS Results of the CP Capacitor after a Certain Duration of Floating (All Values Were Determined Using EC-Lab Software)

CP capacitor after a certain duration of floating	*Q*_dl_ (F s^(α–1)^)	α_dl_	*R*_ct_ (Ω)	*R*_d_ (Ω)	τ_d_ (s)	α_d/M_	*Q*_c_ (F s^(α–1)^)	α_c_
CP – before floating	2.9 × 10^–4^	0.79	0.49	2.07	1.15	0.99	3.55	0.99
CP – 50 h	2.3 × 10^–3^	0.64	0.79	13.5	35.1	0.81	0.89	0.99
CP – 80 h	1.2 × 10^–4^	0.81	112	147	52.1	0.57		
CP – 100 h	8.1 × 10^–5^	0.84	228	227	82.3	0.53		
CP – 140 h	7.1 × 10^–5^	0.81	3929					
CP – 200 h	8.5 × 10^–5^	0.75	18953					

**Table 2:** Values of Individual Components of Electrical Equivalent Circuits Presented in Figure S1 Matched the EIS Results of the VP Capacitor after a Certain Floating Duration (All Values Were Determined Using EC-Lab Software)

VP capacitor after a certain duration of floating	*Q*_dl_ (F s^(α–1)^)	α_dl_	*R*_ct_ (Ω)	*R*_d_ (Ω)	τ_d_ (s)	α_d/M_	*Q*_c_ (F s^(α–1)^)	α_c_
VP – before floating	1.1 × 10^–4^	0.85	0.60	2.39	1.30	0.99	2.35	0.90
VP – 50 h	4.4 × 10^–2^	0.73	0.05	2.15	1.24	1	1442	0.99
VP – 80 h	6.4 × 10^–3^	0.51	0.31	1.32	1.65	0.95	1.32	0.99
VP – 100 h	5.9 × 10^–3^	0.51	0.61	0.91	1.23	0.99	1.21	0.95
VP – 140 h	1.0 × 10^–3^	0.63	0.99	1.42	1.35	0.93	1.67	0.97
VP – 200 h	1.8 × 10^–3^	0.52	5.13	17.4	15.5	0.81	1.21	0.95

Current collectors in electrochemical capacitor systems have direct contact with the carbon electrode materials. On the surface of the current collector, there is the aforementioned passive oxide film composed of iron, chromium, nickel, and molybdenum metal oxides, which is typical for 316 L stainless steel.^51−58^ One of the theories that describes charge conduction in the passive oxide film is the point defect model. It is a theory that assumes that current flows in two specific ways, i.e., through the movement of electrons and electron holes in the solid (i) and as a result of the transport of anions and cations vacancies as well as interstitial cations, i.e., due to the presence of ion conduction (ii). The passive oxide film consists of two layers: an inner barrier and a more porous outer barrier. The outer layer is composed not only of oxides but also, most likely, of hydroxides and oxyhydroxides, especially if the current collector is in constant contact with the electrolyte solution.^51,52^ The mechanism of degradation of steel and metal current collectors is presented in the SI material.^30,31,40,51,52,59^

**Figure 2:**
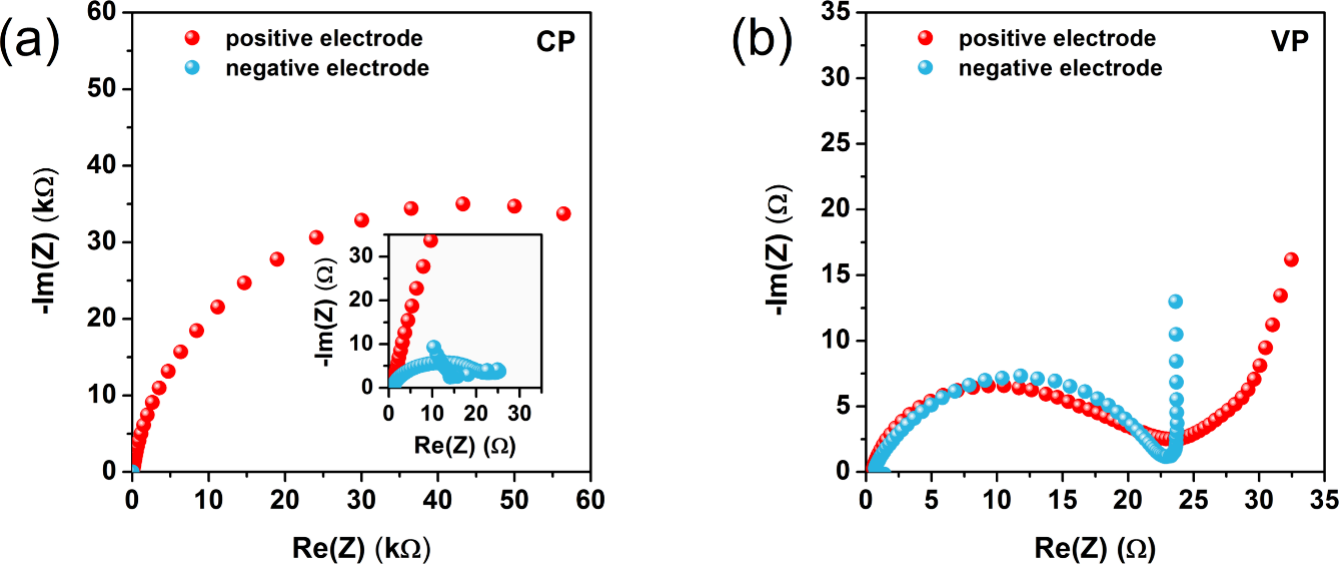
Nyquist plots recorded in stage III (after floating tests) for capacitors operating in (a) constant polarization mode and (b) variable polarization mode. EIS measurements were performed in a three-electrode setup.

In the Nyquist plots for the CP capacitor, the first part of the curve describing the mass diffusion phenomena in the porous space of the carbon material begins to develop (Figure 1c–f). Furthermore, the charge-transfer resistance (*R*_ct_) increases slightly. However, the increase in the *R*_ct_ value is not as pronounced as the increase in the quantities describing the diffusion element M (Table 1). In the fifth cycle of stage II (50 h of floating), the curve begins to lengthen and the slope in the middle frequency range increases. Nevertheless, at this stage, the EEC shown in Figure S1a was used to fit the EIS data, i.e., the same EEC as before floating. At the time of the eighth cycle (80 h of floating), another change in the nature of the curve takes place: an enormous increase in the value of the charge transfer resistance (Table 1). Notably, at this stage, the results of the EIS tests are best described by the EEC presented in Figure S1b. The three-electrode EIS tests of the capacitors CP1, CP2 and VP1, VP2 shown in the SI material confirm that the increase in the charge transfer value is mainly related to the blocking of the porous space in the electrode material and not, as might seem, due to an excessive increase in the thickness of the layer of corrosion products on the surface of the current collector. The complete blockage of this porous space means that diffusion practically ceases to affect the impedance of the tested electrode. Notably, the curves in cycles eighth and 10th (Figure 1d) differ only in the diameter of the semicircle in the high-frequency range, i.e., they differ in the value of the charge transfer resistance. The slope and length of the curve in the middle and low frequency ranges are the same. Therefore, phenomena related to the inhibition of the charge transfer resistance and residual charging/discharging of the electrical double layer at the carbon material/electrolyte interface play a more important role in the range of low-frequency values. The 14th cycle (140 h of floating) indicates complete degradation of the system. The EEC shown in Figure S1c should be used to describe the EIS test results of this and subsequent cycles. The electrical double layer is not charged in any way. At this point, there is complete blockage of the electrode space at the carbon material/electrolyte interface, which is caused by the corrosion products of the current collectors and the activated carbon material.^30,31,40,47^Figure 2a,b shows the results of the three-electrode EIS tests performed in stage III. These tests were carried out 1 week after the last cycle of stage II. The obtained results confirm that positive electrode degradation is responsible for the destruction of the CP electrochemical capacitor system.

The use of variable polarization (VP) extends the lifetime of the electrochemical capacitor system. The shape of the Nyquist curves remains unchanged throughout the entire period of stage I and II measurements (Figure 1g). The values of the individual components constituting the EEC change, but the EEC remains constant over time. All EIS data for the VP capacitor were fitted to the EEC presented in Figure S1a. The use of an optional electrical circuit, i.e., a circuit without a *Q*_c_ parameter element (Figure S1b) makes sense only in the case of cycle 20. However, an EEC containing the *Q*_c_ parameter (Figure S1a) is in this case the best fitted circuit. It is obvious that the values of the elements describing the charge transfer resistance and the diffusion resistance increase with the progress of electrode polarization (Table 2). Each of the electrodes of the VP capacitor plays the role of a positive electrode, and each of these electrodes is subjected to anodic polarization to an equal extent. It was expected that the charge transfer resistance of the individual electrodes of two different capacitors (CP and VP) would differ after long-term tests. The lowest *R*_ct_ value should be characteristic of the negative electrode of the CP capacitor, while the highest value should be characteristic of the positive electrode of the same system. The *R*_ct_ values of both electrodes of the VP capacitor should be, for obvious reasons, similar. However, the three-electrode tests performed in stage III indicate otherwise. As shown in Figure 2, the value of the positive electrode of the CP system is indeed by far the highest, and there is no doubt in this regard, while in the case of the other three electrodes, the *R*_ct_ values are similar. This is probably related to the fact that during the polarization of the negative electrode in the sodium sulfate salt solution, the reduction of oxygen (from air) and water molecules occurs. As a result of these reactions of the oxygen and hydrogen electrodes, the area near the electrodes becomes more alkaline.^53−57^ The concentration of OH^–^ ions increases, which in turn accelerates the reaction of the metal hydroxides and the formation of oxyhydroxides. During the open-circuit voltage intervals (approximately 9 h), i.e., after each cycle of stage II, the negative electrode was in or was approaching the equilibrium state, in which the reduction and oxidation reactions proceeded at the same rate. Therefore, in this case, the formation of a layer of corrosion products, i.e., a layer of hydroxides and oxyhydroxides, could occur. Additionally, hydrogen was released from the negative electrode (CP) throughout the duration of the tests in stages I, II, and III. Due to hydrogen evolution reactions, the Nyquist curve in the medium- and low-frequency ranges may be characterized by the presence of some kind of disturbance. Hydrogen evolution reactions are also the cause of inductive phenomena in such systems.^51,60^ This could also be an indication of ion swapping at the entrance to the pores.

**Figure 3:**
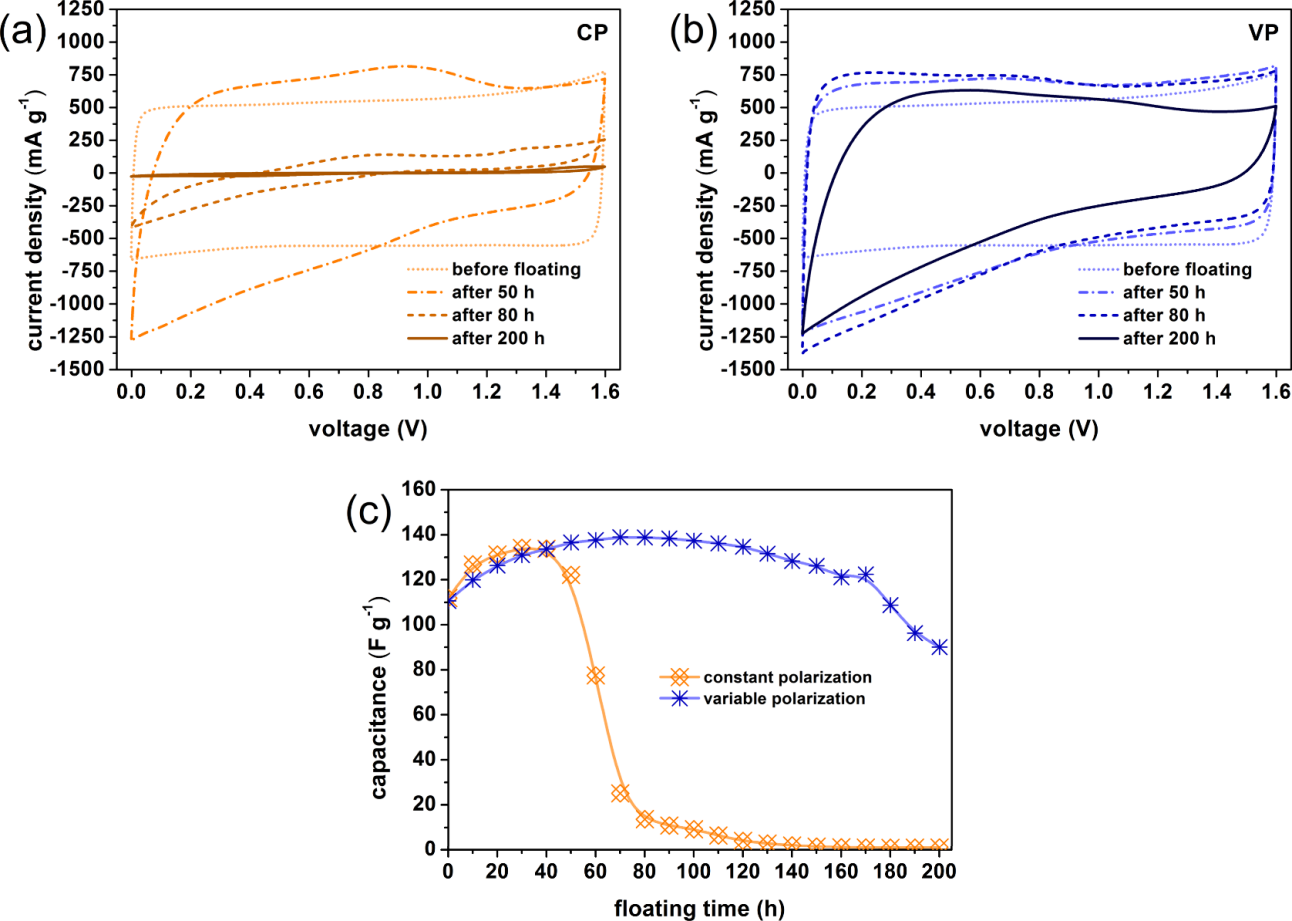
Cyclic voltammograms (a, b) (10 mV s^–1^) and specific capacitance (derived from cyclic voltammetry at 10 mV s^–1^) vs floating time at 1.6 V (c) recorded in stages I and II for capacitors operating in constant and variable polarization modes.

Figure 3a,b shows the results of the two-electrode cyclic voltammetry tests of the CP and VP electrochemical capacitors. These are the results of the analyses carried out in stage I (before floating) and stage II. Stage II involves the long-term floating test procedure described previously. Figure 3c shows the influence of floating on the values of the specific capacitance of electrochemical capacitors, including stage I (0 h).

For the electrochemical capacitor CP, it can be seen that in the initial period of using the long-term test procedure, the specific capacitance of the system increases up to the fourth test cycle (40 h of floating) and then decreases. Nevertheless, in the fifth cycle, the capacitance is higher compared to the characteristics of the system in the first stage of the tests. However, cycle 5 should be considered the first such significant symptom of a decrease in the capacitance value, i.e., the beginning of the degradation of the electrochemical capacitor, which is also indicated by the EIS test results (Figure 1c) described above. The increase in capacitance value at the beginning of stage II is likely related to the transfer of metal ions, which are components of the current collector, to the electrolyte solution, as well as the formation of carbon oxidation products.^30,31,40,47,59^ These ions and smaller particles are actively involved in the charging of the electrical double layer of the electrochemical capacitor. The drop in the capacitance value of the capacitor between the fourth and fifth cycles marks a “breakdown” in the system, i.e., the performance of the capacitor starts to deteriorate rapidly. The value of the specific capacitance drops dramatically until the eighth test cycle, i.e., 80 h of floating. The subsequent drop in capacitance is not as dramatic. It should be remembered that at this point, another change in the nature in the Nyquist curve for the CP system was recorded, that is, the value of the *R*_ct_ resistance increased over a hundred times compared to cycle 5. Moreover, Nyquist curves completely exclude the presence of the *Q*_c_ element, i.e., a parameter describing the charging/discharging of the electrical double layer in the range of low-frequency signal values. It is noted that the continuous and constant decrease in capacitance applies to the period of floating between 80 and 140 h. Afterward, another “breakdown” follows; after this time, the decrease in capacitance becomes less noticeable once again, which is consistent with another change in the nature of the Nyquist curves. It should be noted that the CP electrochemical capacitor lost its usefulness after approximately 50 h of floating; however, the 14th cycle can be considered the cycle when this system ceases to fulfill its function completely. In comparison, the values of the specific capacitance of the electrochemical capacitor with variable polarity (VP) were higher in stage II than in stage I up to the 18th test cycle (180 h of floating).

The last part of the electrochemical capacitor studies (stage IV) is provided and discussed in the SI material (Figure S2a–d).

**Figure 4:**
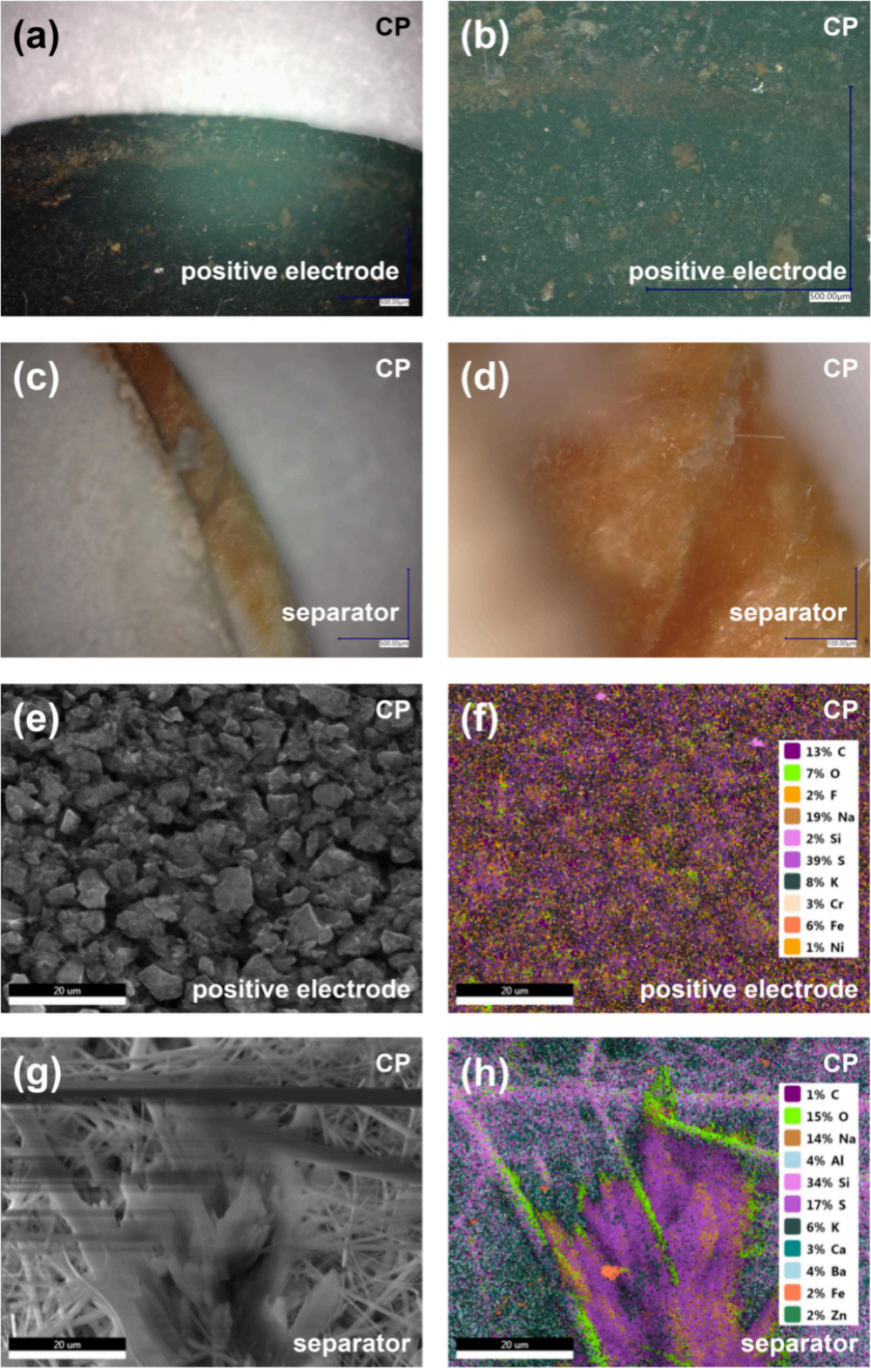
Keyence microscope images (a–d), scanning electron microscope images (e, g) and energy-dispersive X-ray spectroscopy maps (f, h) of the positive carbon electrode and separator from the CP capacitor after 200 h of floating.

Figure 4a–d presents images taken with the Keyence microscope. It shows the surface of the positive carbon electrode and separator of the CP capacitor. The images indubitably indicate the presence of rust on both surfaces. Nevertheless, to check the components of the current collectors at a certain depth of these elements, EDS tests were performed. The so-called clean areas, i.e., untouched by the presence of a brown deposit, were tested. Figure 4f,h shows the EDS mapping area of the separator and carbon surfaces. The obtained result reflects the state at a depth of more than 1 μm into the structure and clearly shows that components of stainless steel, i.e., iron, chromium, and nickel, are absorbed onto the EC electrode material and the separator. The presence of other metals in the separator (e.g., zinc) is a result of the material composition. In addition, each of the tested samples was not washed with distilled water to avoid the potential removal of corrosion products, which are not bound to the substrate. Hence, there are components of the electrolyte solution on the maps showing the surfaces. Unlike the above materials, the steel elements were thoroughly washed in an ultrasonic cleaner and dried after aging tests.

**Figure 5:**
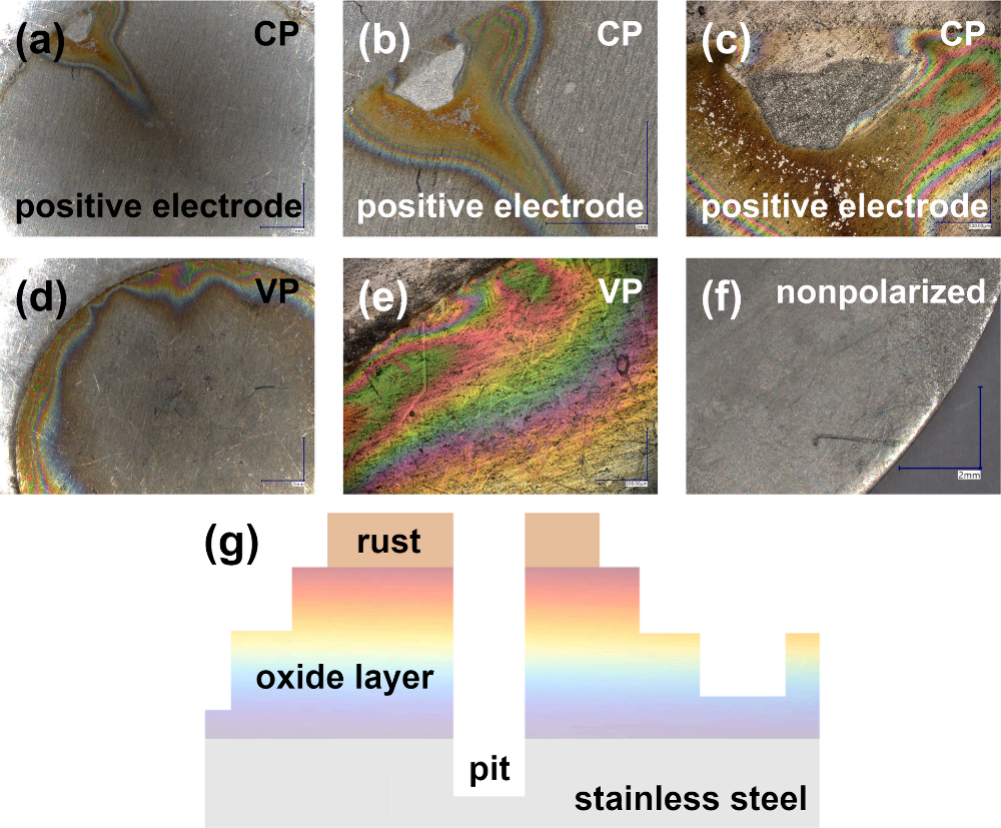
Keyence microscope images of 316 L stainless steel discs subjected to constant anodic (a–c) polarization, variable (d, e) polarization, no polarization (bare steel surface) (f), and schematic cross section (g) of the 316 L stainless steel current collector (of positive electrode) surface after severe corrosion in the electrochemical capacitor.

The penetration of steel ingredients into the porous space of the carbon electrode material is confirmed by the physicochemical analyses of the steel surface that was previously subjected to a long-term aging procedure according to the stage II regime for capacitors. Figure 5a–f shows images of 316 L stainless steel discs taken with a Keyence microscope. These are images of the unmodified (nonpolarized) surface and the surface subjected to constant (CP) and variable (VP) polarization for a period of 80 h, i.e., after the CP capacitor was completely degraded, losing slightly more than 90% of its initial capacitance, while the VP capacitor was still working flawlessly, almost reaching its maximum capacitance value. Several issues are noticeable in the images presented. First, the surface of the steel marked as VP appears to be more damaged by corrosion compared to that of the CP sample. The presence of rainbow colors indicates an increase in the thickness of the oxide layer during polarization to dimensions in the wavelength range of visible light, that is, 400–1000 nm.^61^ As already mentioned, when comparing the CP and VP samples, in the case of the latter, most of the surface is covered with an oxide with a thickness corresponding to the wavelength range of visible light. On this basis, it can be initially concluded that the oxide layer formed on the surface of the VP steel is generally thicker than the layer on the CP steel. In addition, the capacitor tests and the three-electrode tests, shown later in the manuscript, indicate that the CP sample exhibits inferior anticorrosive properties. This is certainly due to the presence of a huge pit on the surface of the CP sample. Constant polarization not only caused the buildup of an oxide layer but also resulted in the breakage of this layer at some point and induced iron oxide (rust) formation. Notably, the surface around the pit is surrounded by a thick oxide layer. Rust, which is a product of oxidation of the steel surface at the bottom of the pit, is closest to the edge of the pit. The pitting edge is then the highest point on the sample topography. Below is the oxide, the thickness of which is within the wavelength of visible light, i.e., the thickness of the oxide layer decreases as the distance from the pitting edge increases. Figure 5g shows a schematic of the pit cross section together with the layer of corrosion products (rust). Note that this represents a deep-polarization corrosion mechanism for steel. In fact, the thickness of the oxide layer does not need to gradually decrease as the distance from the edge of the pit increases. Scanning electron microscopy and atomic force microscopy analyses are provided in the SI material (Figures S3a–c and S4a–f) to supplement and complement the data on 316 L steel surface corrosion.

**Figure 6:**
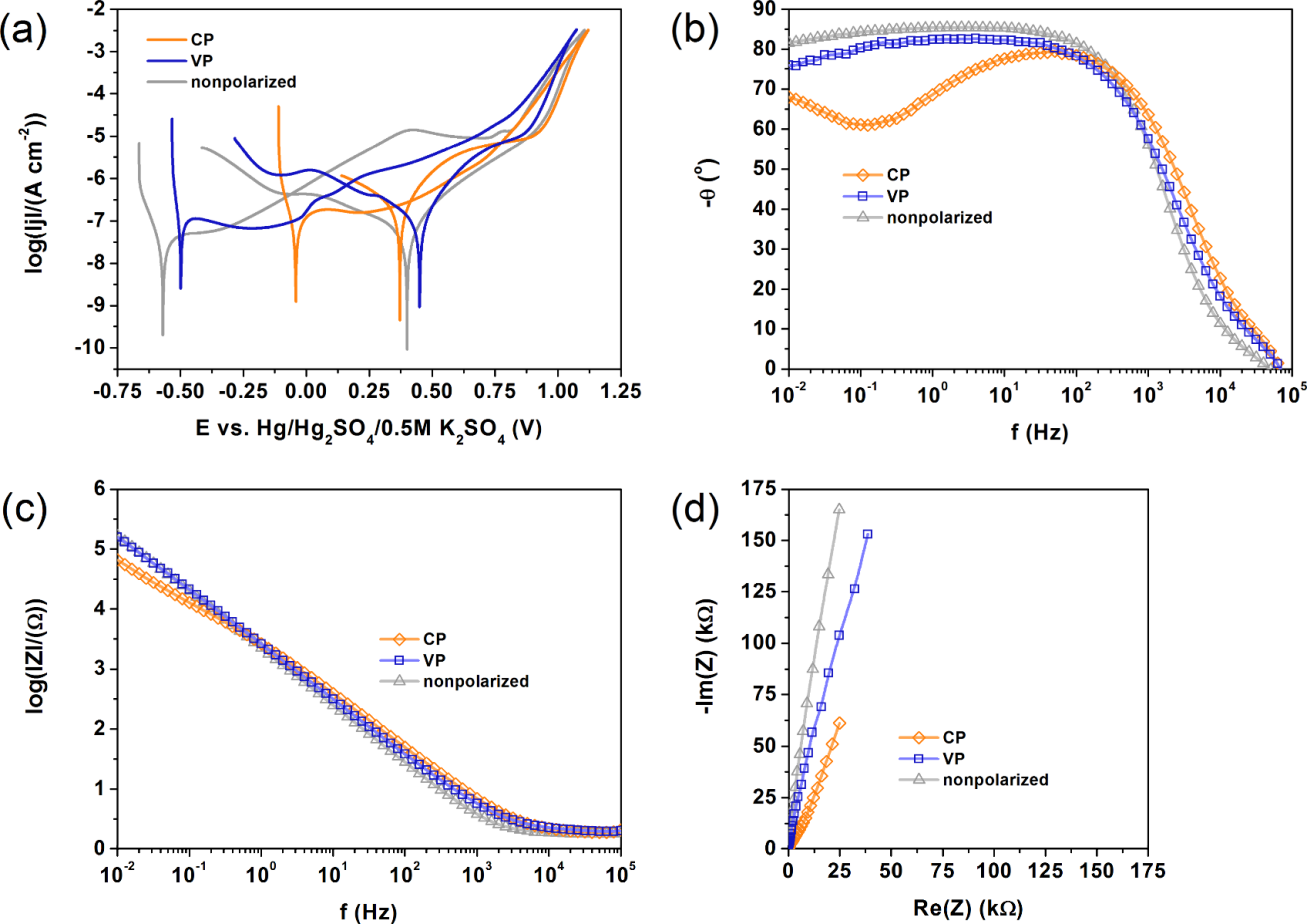
Cyclic potentiodynamic polarization (a) and electrochemical impedance spectroscopy (b–d) test results of nonpolarized 316 L stainless steel discs and steel discs after strong anodic polarization (according to stage II) in constant and variable modes.

The reason for the destruction of the current collectors in both types of electrochemical capacitors is very well illustrated by the results of three-electrode cell measurements of the three aforementioned steel samples presented in Figure 6a–d and in Table 3. Cyclic potentiodynamic polarization (CPP) tests indicate that the unmodified sample has the lowest corrosion current density (*j*_corr_), which is expected.^30,31,40^ The corrosion current density (*j*_corr_) values for both modified samples are much higher, with the highest value in the CP sample. The influence of the types of polarization is much more noticeable in terms of the shape of the potentiodynamic curves and the presence of the so-called secondary passivation peaks.^52,62,63^ Although the oxygen evolution potential is more or less similar for all samples, the potential range from corrosion potential (*E*_corr_) to oxygen evolution potential (*E*_OE_) is the widest for unmodified and VP samples. Therefore, in a working capacitor that has been subjected to constant polarization, the decreasing range of the positive electrode current collector potential may cause intense oxygen evolution on its surface, i.e., faster electrolyte decomposition. On the other hand, released oxygen is a substrate for the formation of oxidized carbon compounds, that is, the corrosion of carbon materials presented by Piwek et al.^47^

**Table 3:** Corrosion Potentials (*E*_corr_), Corrosion Current Densities (*j*_corr_), and Oxygen Evolution Potentials (*E*_OE_) of All Tested Samples (Values Were Estimated on the Basis of the Tafel Method Using EC-Lab Software)

sample	*E*_corr_ (mV)	*j*_corr_ (nA cm^–2^)	*E*_OE_ (mV)
nonpolarized	–569	43	1106
CP	–42	98	1121
VP	–499	79	1074

As already mentioned, the occurrence of carbon corrosion cannot be ruled out. Moreover, on the basis of the above hypotheses, this phenomenon can be presented as a consequence of the corrosion of the current collector during deep anodic polarization of the positive electrode. It is also worth noting the dual nature of the electrode polarization curves. Unmodified steel (nonpolarized), apart from the lowest corrosion current density value, also shows the presence of three peaks in the range of anodic polarization. Modified samples are characterized by the presence of only the last peak, which can undoubtedly be ascribed to phenomenon related to oxygen evolution. The two previous peaks are related to chromium (first peak) and most likely nickel and/or manganese (second peak) oxidation reactions.^52^ While the origin of the first peak is quite obvious, the above interpretation of the second peak may be slightly questionable. Therefore, the mechanism of 316 L stainless steel corrosion and transpassivation phenomenon under deep anodic polarization is extensively discussed in the SI material.^31,32,40,51−58,64−69^

**Figure 7:**
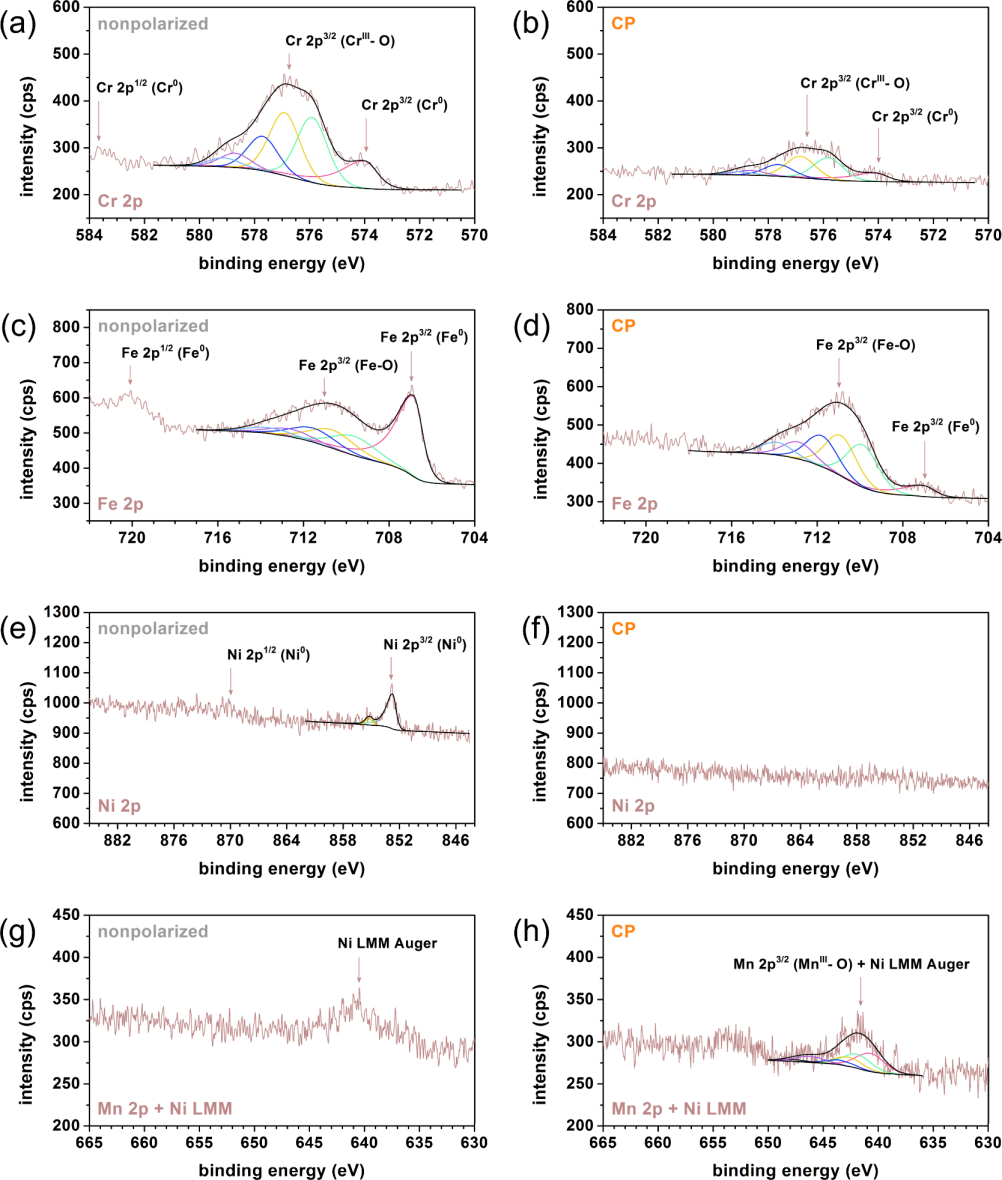
High-resolution XPS spectra of nonpolarized 316 L stainless steel disc (a, c, e, g) and steel disc after strong anodic polarization (according to stage II conditions) in constant mode (b, d, f, h).

On the basis of the obtained results, it can be concluded that with the progress of anodic polarization, i.e., with each subsequent cycle of the aging procedure, the oxide layer becomes thicker. At some point, this layer breaks, and pitting begins. In the case of the CP sample, this phenomenon is faster because the forces acting on the oxide layer are greater. The XPS physicochemical analysis (Figure 7a–h and Table 4) is complementary to the three-electrode electrochemical tests for three different samples of stainless steel. However, discussion in this matter is given in the SI material. Near the corrosion potential, that is, around the steady state, the corrosion current density values are the highest for steel samples subjected to the aging procedure. This is due to the presence of pits, i.e., discontinuities in the oxide coating. The passive oxide layer present on the surface of unmodified steel is a semiconductor that effectively protects the surface of the steel.^51−58^ Under the conditions of strong anodic polarization, i.e., during significant disturbances of the electrode from the steady state, the current density increases. However, the anodic current in this case is lower for the modified samples. This is related to the presence of nonreactive chromite on the surface of CP and VP steel. Then, a large part of the current flows through the pits, which are iron channels and facilitate the formation of rust around the pits, i.e., in locations that have the highest degree of surface oxygenation.^53−57^

**Table 4:** Contribution (%) of Elements (without Carbon) to the Subsurface of 316 L Stainless Steel before (Nonpolarized) and after (CP) Long-Term Aging (Results Were Derived from X-ray Photoelectron Spectroscopy Analysis)

elements		nonpolarized steel	CP
	Fe^0^	8.0	1.7
Fe	Fe–O	9.1	17.1
	Fe	17.1	18.8
	Cr^0^	2.8	1.0
Cr	Cr^III^–O	13.2	5.5
	Cr	16.0	6.5
Ni		3.3	0.0
Mo		1.1	0.0
Mn		0.0	5.8

**Figure 8:**
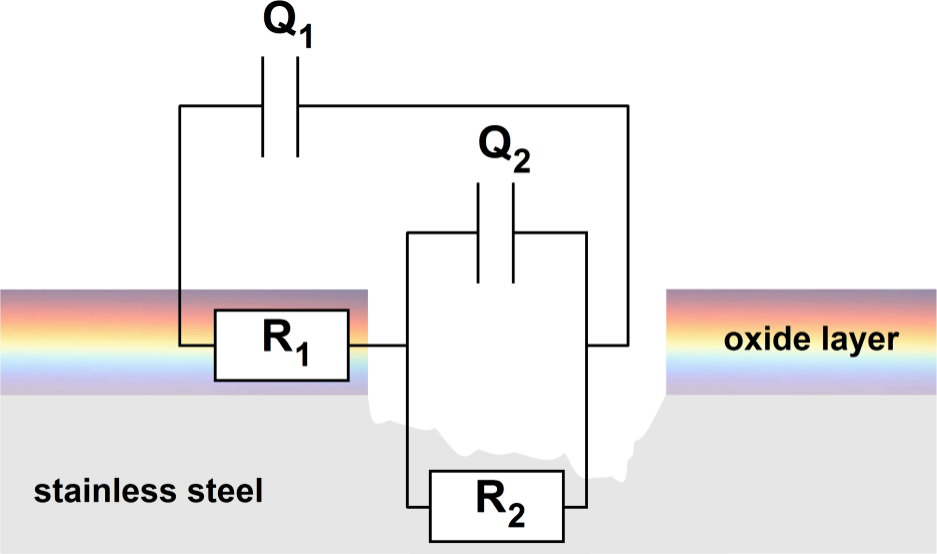
Electric equivalent circuit that could be used to describe 316 L stainless steel after pitting.

In addition, the newly created rust enters the electrolyte solution and, in the case of electrochemical capacitors, into the porous space of the carbon electrode material. The differences in the electrochemical characteristics of steel within the corrosion potential and in the area of deep anodic polarization are also revealed by electrochemical impedance spectroscopy (EIS). The EIS tests were performed with a low-amplitude signal vs open circuit potential. The results are presented in Figure 6b–d in the form of Bode and Nyquist plots. The dependence of the phase angle on the frequency indicates the presence of a time constant that describes the electric double layer at the electrolyte/oxide layer interface for the unmodified and VP samples.^51,60,70^ In the case of CP steel, there is a second time constant (the second loop) in the range of low frequency values, which reflects the lower coverage of the steel surface with an oxide layer, i.e., the presence of pits. As a result, the value of the impedance modulus at the lowest frequency value is the lowest for CP steel (Figure 6c). The second time constant corresponds to the formation of an electrical double layer at the 316 L stainless steel/electrolyte interface. The electrical double layer at both types of interfaces can be described in the usual way by the *Q*_dl_ parameter and *R*_ct_, as presented in Figure 8.

**Figure 9:**
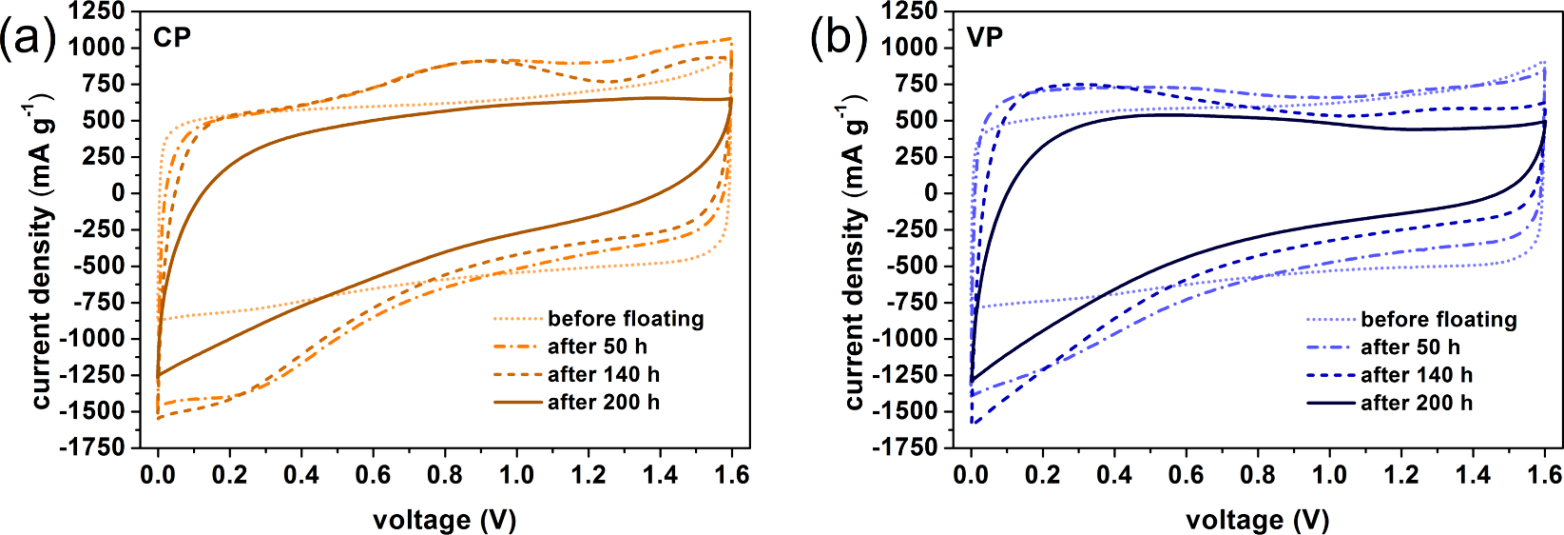
Cyclic voltammograms (10 mV s^–1^) recorded in stages I and II for capacitors with Au current collectors operating in (a) constant polarization mode and (b) variable polarization mode.

**Figure 10:**
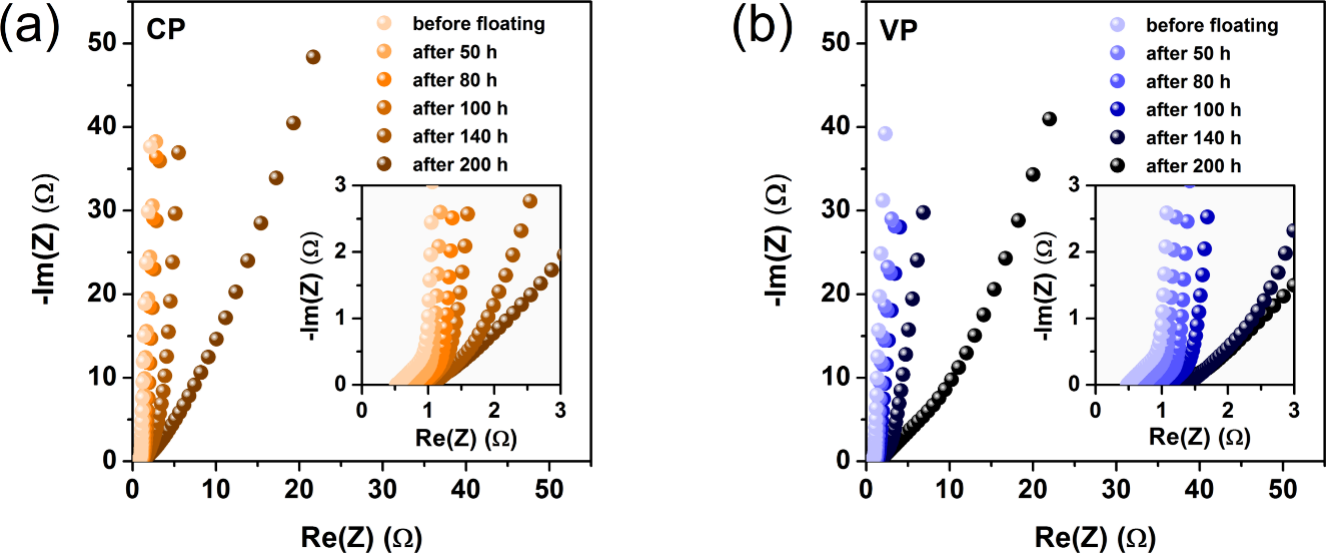
Nyquist plots recorded in stages I and II for capacitors with Au current collectors operating in (a) constant polarization mode and (b) variable polarization mode.

**Figure 11:**
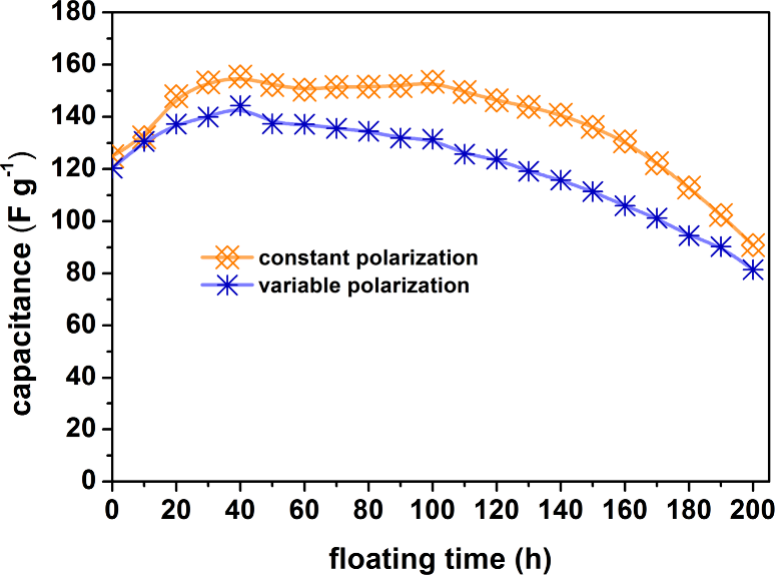
Specific capacitance (derived from cyclic voltammetry at 10 mV s^–1^) vs floating time at 1.6 V for capacitors with Au current collectors operating in constant and variable polarization modes.

The results of the accelerated aging procedure for electrochemical capacitor systems containing gold current collectors confirm all of the presented hypotheses. Figures 9a,b and 10a,b show the curves for cyclic voltammetry and electrochemical impedance spectroscopy. The graph of capacitance as a function of the duration of the potentiostatic method (Figure 11) presents a slow degradation of the capacitor system, markedly slower than in the case of a capacitor with steel collectors. Gold does not corrode at the rate of stainless steel, so there are no corrosion products that could clog the pore space of the carbon electrode material.^53−57^ In this case, the slow degradation of the system is associated with the decomposition of the electrolyte and corrosion of the carbon material. However, these processes are also much slower than in the case of steel collectors. The most surprising result of these tests is the fact that the capacitance values of the three different capacitors, i.e., two with gold collectors and the VP one, are very similar to each other after 200 h of floating.

## 4 Conclusions

The results of the conducted research lead to one important conclusion. The factors causing the degradation of electrochemical capacitors, i.e., the corrosion of the carbonaceous material and the decomposition of the aqueous electrolyte solution, originate from the electrochemical corrosion of the current collector of the positive electrode. Strong anodic polarization causes pitting on the surface of the passive stainless steel oxide film and consequently the formation of solid corrosion products, mainly rust, which enter the electrolyte solution and block the pore space of the carbon material. In addition, the destruction of the passive layer causes a narrowing of the range of the working potentials of the positive electrode, which increases the intensity of oxygen evolution from electrolyte solution (electrolyte decomposition) and consequently the corrosion of the carbon material. The use of a variable polarization mode allows for an extended life in the electrochemical capacitor because it slows the corrosion of the current collector, thereby delaying aqueous electrolyte decomposition and carbon corrosion.

## Supporting Information Available

Electrical equivalent circuits used for fitting the EIS data of electrochemical capacitors (Figure S1); meaning of parameters Q, R, and M (description); mechanism of degradation of steel current collectors in aqueous electrolyte solutions (description); electrochemical measurements in stage IV (description and Figure S2); scanning electron microscopy and atomic force microscopy analysis of 316 L stainless steel after constant (anodic) and variable polarization (description and Figures S3 and S4); mechanism of 316 L stainless steel corrosion and transpassivation phenomenon under deep anodic polarization (description); references relating to the contents of the file (PDF)
