# Supplementary material for: Interfacial Insights into the Polarization Protocol: Toward Reducing Corrosion and Improving the Cycle Life of Electrochemical Capacitors
Source: ACS Appl Mater Interfaces. 2024 May 18;16(21):27242–53. doi: 10.1021/acsami.4c00767 (PMC11145588; doi:10.1021/acsami.4c00767)
Supplement: Supplementary file 1 — am4c00767_si_001.pdf [file am4c00767_si_001.pdf]

SUPPORTING INFORMATION

Interfacial Insights into the Polarization Protocol: Toward Reducing Corrosion and Improving the Cycle Life of Electrochemical Capacitors

Jarosław Wojciechowski,*[a] Katarzyna Szwabińska,[b] Krzysztof Fic*[a,c] and Grzegorz Lota*[a,c]

[a] Institute of Chemistry and Technical Electrochemistry, Poznan University of Technology, Poznan 60-965, Poland. E-mail: jaroslaw.g.wojciechowski@put.poznan.pl, krzysztof.fic@put.poznan.pl, grzegorz.lota@put.poznan.pl

[b] Faculty of Chemistry, Department of Inorganic and Analytical Chemistry, Electrochemistry@Soft Interfaces Team, University of Lodz, Lodz 91-403, Poland.

[c] Łukasiewicz Research Network – Institute of Non-Ferrous Metals Division in Poznan, Central Laboratory of Batteries and Cells, Poznan 61-362, Poland.

Electrical equivalent circuits

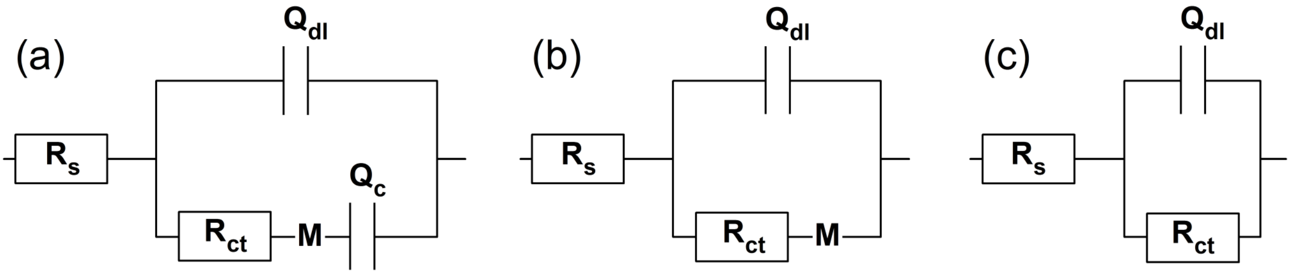

Figure S1. Electrical equivalent circuits (EECs) used for fitting the EIS data of electrochemical capacitors. (a) EEC composed of R_s, R_{ct}, M, Q_{dl}, Q_c elements, (b) EEC composed of R_s, R_{ct}, M, Q_{dl} elements and (c) EEC that includes R_s, R_{ct}, Q_{dl} elements only.

Meaning of parameters Q, R and M

As a reminder, note that the Q parameter is equivalent to a nonideal capacitor element. It is applied in systems where there is capacitance dispersion as a result of the chemical heterogeneity of the electrode material surface. The values of the Q parameter are expressed in F $s^{(\alpha-1)}$, which is consistent with the impedance formula of the constant phase element (CPE) given by Equation (1):

$$Z_{CPE}(j\omega) = \frac{1}{Q(j\omega)^{\alpha}} \qquad (1)$$

where j and ω are an imaginary unit and an angular frequency, respectively, while α is a factor reflecting the measure of capacitive dispersion ($0.5<\alpha<1$).[1] In the case of capacitor systems,

elements Q, R and M describe the electrolyte resistance R_s, an electric double-layer in the high frequency range Q_{dl} (semicircle), the charge transfer resistance R_{ct} (semicircle), diffusion, or rather a special case of diffusion, i.e., restricted linear diffusion (M), which is illustrated by a straight line with a slope equal to (-1) in the range of medium frequency values and an electric double-layer Q_c in the range of low frequency values (straight, vertical line).

As already mentioned, both parameters Q_{dl} and Q_c describe an electrical double layer at the electrode/electrolyte interface. However, in the first case, it concerns the faradaic reactions and the associated charge-transfer resistance R_{ct} at the carbon electrode/electrolyte interface and at the current collector/carbon electrode interface. The Q_c parameter describes the formation of an electric double layer at the carbon electrode/electrolyte interface without the participation of faradaic reactions, i.e., in this case, the capacitor charging/discharging cycle takes place, and the value of the Q_c parameter no longer depends on the frequency. This is because at a lower frequency value, the electric signal is capable of penetrating deeper into the porous space of the carbon material.[2]

In previous work on the corrosion of stainless steel surfaces protected by siloxane coatings, the use of the M element was associated with the occurrence of mass diffusion and, more precisely, with the formation of a layer of corrosion products under the siloxane coating, which is a porous coating that is to some extent permeable to aqueous electrolyte solutions.[3-5] The described corrosion layer consisted of metal hydroxides, oxides, metal oxyhydroxides, and an electrolyte solution. Its location between the passive oxide film on the 316 L stainless steel surface and the siloxane coating indicates the effect of restricted linear diffusion with reflective boundary conditions. Generally, the M element refers to diffusion conditions when a concentration gradient does not form in the diffusion layer, i.e., charge transfer across the interface does not exist or is extremely slow. Therefore, considering the negligible number of faradaic reactions at the carbon material/electrolyte interface, the presence of this element in EDLCs is fully justified. The impedance of the M element is described by Equations (2) and (3). The latter includes the α coefficient, that is, a coefficient that measures deviation from the ideal state of the described phenomenon, as in Equation (1):

$$Z_{\mathrm{M}}(j\omega) = R_d \frac{coth\,(\sqrt{\tau_d j\omega})}{\sqrt{\tau_d j\omega}} \quad (2)$$

$$Z_{M}(j\omega) = R_d \frac{coth\left((\tau_d j\omega)^{\frac{\alpha}{2}}\right)}{(\tau_d j\omega)^{\frac{\alpha}{2}}} \quad (3)$$

R_d and τ_d are the diffusion resistance (Ω) and the diffusion time constant (s), respectively. τ_d is equal to L^2/D, where L and D represent the thickness of the diffusion layer and the diffusion coefficient, respectively.[6-8] The use of elements Q, R, and M is in this case related to the nature of phenomena that occur in such systems.[6-10]

Mechanism of degradation of steel current collectors in aqueous electrolyte solutions

In electrochemical capacitor systems, the current collector forms two interfaces, i.e., with the electrolyte solution and the carbon material. This is due to the presence of discontinuities and damage of the carbon electrode material and the binder. Under equilibrium (stationary) conditions, the passive film is consumed and rebuilt simultaneously.[10,11] It is related to the equal rates of the dissolution reaction of the passive oxide film and the reduction of oxygen molecules on its surface. During charging, the positive current collector oxidizes in constant contact with the electrolyte solution and the carbon material. As the time in the charged state increases, the current collector of the positive electrode gradually degrades and the passive film dissolves. The outer part of the passive oxide film, i.e. the more porous part composed of oxides, hydroxides, oxyhydroxides, salts of the current collector components (316 L stainless steel) becomes thicker. At some point, these compounds begin to move into most of the electrolyte solution. In the beginning, they are mainly metal ions that are actively involved in the charge of the electrical double-layer.[12] The metal compounds appear in the electrolyte solution, blocking the porous space of the carbon material and newly created oxidized carbon species. This is indicated by the progressive changes in the nature of the Nyquist plots for the CP capacitor.[3,13,14]

Electrochemical measurements in stage IV

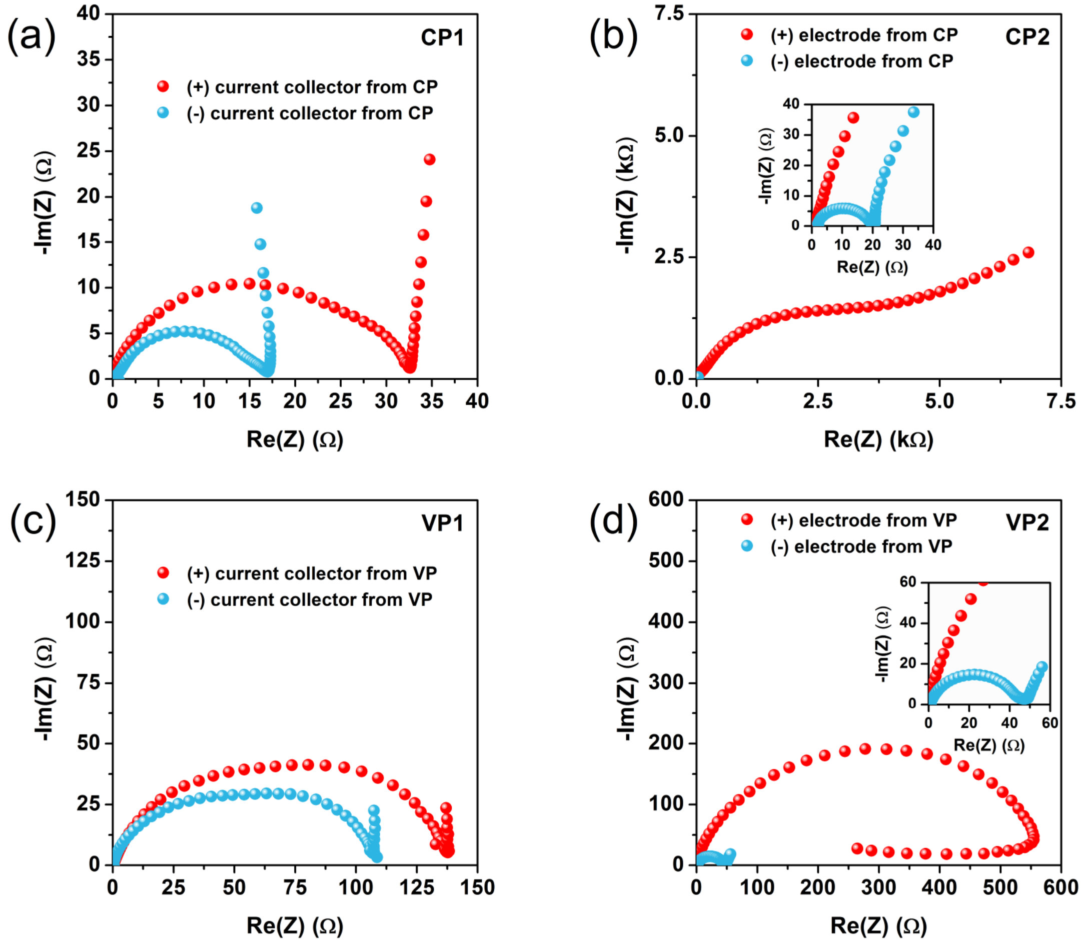

Figure S2. Nyquist plots recorded in stage IV (i.e. after all floating tests) for capacitor assembled from (a) fresh carbon electrodes and current collectors from capacitor CP after stage III, (b) fresh current collectors and carbon electrodes from capacitor CP after stage III, (c) fresh carbon electrodes and current collectors from VP capacitor after stage III, and (d) fresh current collectors and carbon electrodes from capacitor VP after stage III. EIS measurements were performed in a three-electrode setup.

Figure S2a-d shows the results of three-electrode impedance tests of four different capacitors, i.e. CP1, CP2, VP1 and VP2. As already mentioned in the experimental section, these are systems that included fresh carbon electrode materials, soaked in a freshly prepared electrolyte solution and current collectors used earlier in the previous stages (CP1 and VP1), as well as used electrode material and new, freshly prepared current collectors (CP2 and VP2). It should be noted that the results obtained in this way may not always be reliable, i.e. they will not always reflect the real picture of the situation. This is due to the fact that after 200 h of floating the system is in a specific state of equilibrium, in which certain phase boundaries

are established between the electrolyte, carbon material, current collector, and corrosion products. Separating the electrode material with the separator from the surface of the current collector, even as carefully as possible, will not ensure complete reproduction of the conditions and phenomena that occur in each of the capacitor components prior to decomposition, especially when the carbon electrode material undergoes some mechanical damage. Additionally, one should not forget about the irretrievable loss of the layer of loose products on the surface of the extensive layer of corrosion products of the current collector, i.e., in the vicinity of the carbon electrode material. However, the results obtained allowed for quite unequivocal conclusions to be drawn. When describing the results presented in Figure S2a-d, it is also worth mentioning a certain regularity. On the basis of the previous results, it can be concluded that the charge transfer resistance for the steel collector is much greater than for the porous carbon material. Therefore, electrochemical capacitor systems in which carbon material is deposited on the surface of the current collector are characterized by lower charge transfer resistance values. It is the result of a very well-developed specific surface of activated carbon, which is an electrically conductive material. When comparing the two types of systems, i.e. 1 and 2, it is noted that the carbon electrode materials had the greatest impact on the degradation of the capacitor systems. An ideal example of this, i.e. the most expressive and authoritative, is the capacitor CP, the electrodes of which were subjected to constant, fixed polarization. The positive electrode of the CP2 system is completely destroyed. Exactly the same electrode for the CP1 capacitor is also characterized by a much higher R_{ct} value compared to the results of the tests in stage I, or at the beginning of stage II. On this basis and on the basis of the results of EDS analysis (presented in the manuscript) and AFM analysis (presented in the further part of the ESI material), it is concluded that the corrosion products of the current collectors mostly penetrate into the porous space of the carbon electrode material, thus leading to the loss of its functionality and degradation of the entire electrochemical capacitor system. To a much lesser extent, corrosion products appear on the surface of the current collector. Nevertheless, it is also an extremely significant phenomenon, because when using the collectors from stage I and II the resistance values are almost 20 and 40 ohms for the respective electrodes (Figure S2a). Quite an important issue, and indirectly confirming the fact of the passage of corrosion products into the space of the carbon material and the separator, are the differences in the R_{ct} values for individual systems containing degraded current collectors (CP1 and VP1) (Figure S2a and Figure S2c). In this case, the electrodes of the VP1 capacitor show much higher values, which proves that the layer of corrosion products on the surface of the collectors is thicker. This is in line

with the results of the stage III three-electrode study mentioned earlier (Figure 2). The similar values of the charge transfer resistance for the three electrodes: negative (CP), positive (VP), and negative (VP) prove that in this case the amount of corrosion products in and on the carbon material is much lower than for the positive electrode of the CP capacitor. The layer of corrosion products on the surface of the current collectors plays a significant role in this situation. The result of the impedance test for the positive electrode of the VP2 capacitor should certainly not be treated as an error. In this case, it is important to know the configuration in which the test was completed. Well, the electrode marked as positive was in fact polarized anodically during the last floating test. The Nyquist curves for the electrodes of the VP1 capacitor show two very poorly separated semi-circles in the high frequency range. These two semicircles actually form one describing the layer of corrosion products. However, such a curve outline allows us to believe that the layer in question consists of two nearly identical layers (two almost identical time constants). These can be layers of solid and hydrated products, as mentioned above.

Scanning electron microscopy and atomic force microscopy analysis of 316 L stainless steel after constant (anodic) and variable polarization

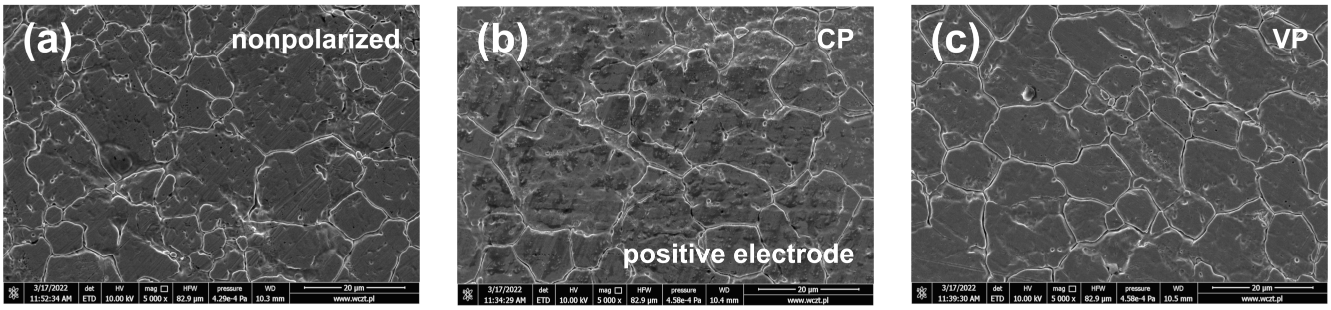

Figure S3. Scanning electron microscope images of (a) bare (nonpolarized) 316 L stainless steel disc surface and steel discs surfaces after strong polarization: (b) constant anodic and (c) variable.

Figure S3a-c shows SEM pictures of the surface of unmodified steel, CP, and VP samples (Figure 5). The images were taken in the center of the surface, which had been polarized for 80 hours. For the eye without a microscope, it is an intact area, i.e. an area without rust or rainbow deposits, similar to that for all three tested samples. The SEM images presented are also practically the same. In this magnification, each sample has an almost identical surface. The only difference seems to be the greater surface roughness of CP steel, i.e. steel subjected to more severe long-term polarization, which is confirmed by the results of the AFM tests. At this point, it is worth emphasizing that the surface of the 316 L stainless steel samples

obtained from the manufacturer had a 2B finish according to the specification nomenclature. It is one of the most common surface preparations for austenitic stainless steels.[15-18] In this case, the elements are cold rolled, annealed, pickled, and finally rolled with polishing rolls. Treatment carried out in this way results in a gray surface intended for further decorative processing. The images shown in Figure S3a-c, i.e., the grain structure of the steel surface is the result of the annealing and cooling process of the steel.[19] Nevertheless, the surface provided by the manufacturer has a passive oxide layer, i.e., a protective layer, the thickness of which does not exceed 10 nm. It is a layer invisible to a scanning electron microscope and an atomic force microscope.[20] As already mentioned, the 2B finish allows for further surface treatment e.g. grinding to obtain a mirror-like surface. However, the anti-corrosive properties of ground stainless steel are extremely poor compared to the starting product obtained from the manufacturer. This is most likely due to the removal of some of the steel material on which the protective passive layer was formed. To support this hypothesis, scratches were made on the surface of all three stainless steel samples with a steel lancet. Three cross-sectional profiles were then made through the designated crevices. The AFM pictures of the surface of the tested samples and the crevices depth histograms are presented in Figure S4a-f.

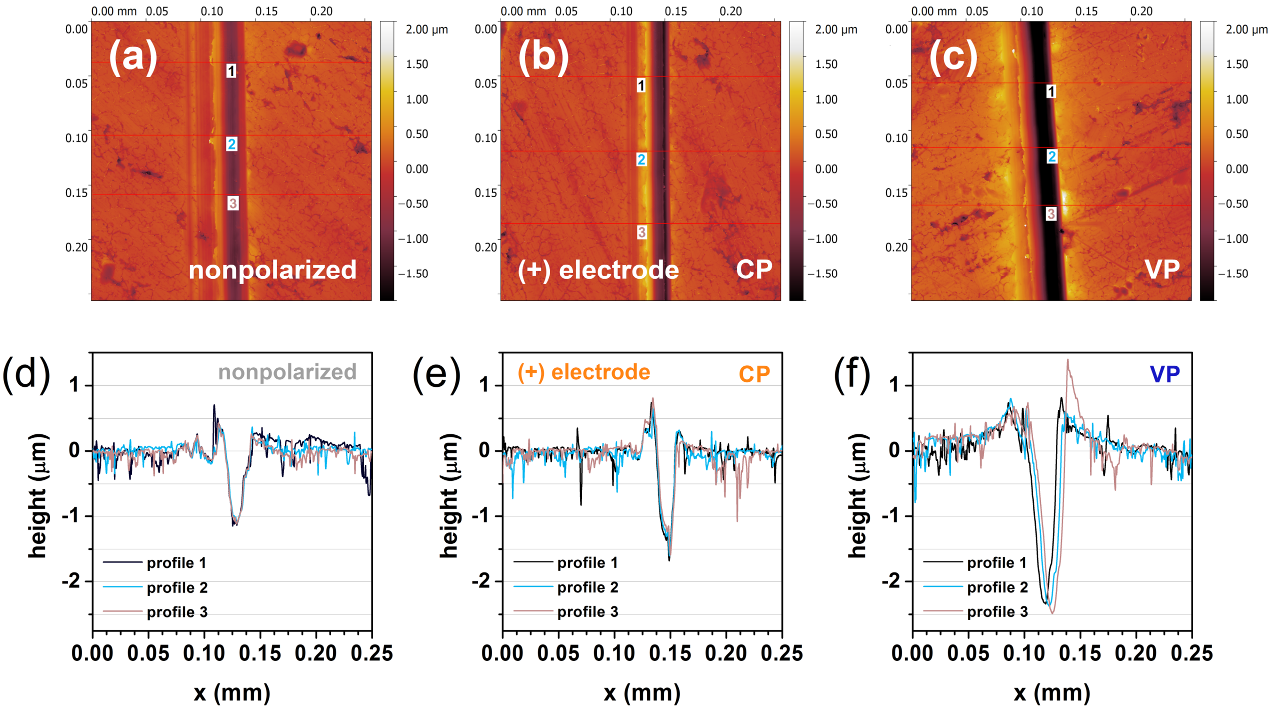

Figure S4. Atomic force microscope images of (a) bare (nonpolarized) 316 L stainless steel disc surface and steel discs surfaces after strong anodic polarization: (b) constant and (c) variable. All of the surfaces were scratched with a stainless steel lancet, while depth histograms of the resulted crevices are presented in plots (d–f).

At this point, attention should be paid to the roughness values of the tested samples, which were equal to 0.1077 μm (bare sample), 0.1206 μm (CP) and 0.1028 μm (VP). In the case of unmodified steel and steel marked as VP, these values were similar and definitely the lowest in comparison to the sample after constant polarization (CP) mode, which is consistent with the SEM images. The penetration of the lancet into the VP sample is the deepest, which once again confirms the fact that the layer on the variably polarized sample is the thickest. Previous evidences, supporting this hypothesis, were greater surface coverage with rainbow-colored oxide, as visualized with the Keyence microscope, and the results of CP1 and VP1 electrochemical capacitors research (Figure S2a and c). In the last case, the results of impedance tests confirmed that the charge-transfer resistance is much higher in the case of current collectors from the VP capacitor.

Mechanism of 316 L stainless steel corrosion and transpassivation phenomenon under deep anodic polarization

The results of the XPS tests presented in Figure 7a-h seem to confirm the presence of the oxidation reaction of nickel or manganese on the stainless steel surface. Furthermore, Table 4 shows the contribution (%) of individual elements in the tested area. The results obtained reflect the subsurface situation of stainless steel, i.e. at a depth not exceeding 10 nm. Therefore, it is possible to characterize the passive oxide layer on the surface of the unmodified sample. Note that the results presented do not contain carbon, which occupied the largest part of the surface. In the case of stainless steel, the presence of this element is not surprising, which has been confirmed so far many times.[21-23] Its origin is mainly due to two factors. These are the presence within the grain boundaries, which are perfectly visible in the SEM images (Figure S3a-c) and the atmosphere contamination.[21-23] In the first case, an enormous amount of carbon accumulates in crevices as a result of sensitization of the steel surface, i.e. after the annealing and then cooling processes. To make the surface composition with metal oxides more transparent, the carbon content has been removed. On the basis of the obtained results, it is concluded that the greatest part of the surface is occupied by oxygen in the form of iron, chromium, nickel, and molybdenum oxides. This is in line with the literature.[10,11,24-29] Moreover, the presence of iron, chromium, nickel, and molybdenum cations in the given amounts is also not surprising. The existence of a passive oxide layer on the surface of stainless steel is described by several theories, including the point defect model, which is the most

popular.[10,11] According to this theory, the passive layer is a highly distorted layer through interstitial inclusions and through cationic and anionic vacancies. Layer formation consists of the transport of metal cations from the steel bulk to the passive layer and the transport of oxygen ions from the electrolyte solution. There is also the phenomenon of transporting metal ions from the passive layer to the electrolyte solution without changing their oxidation state. Nevertheless, under equilibrium conditions, the thickness of the passive layer increases if the reaction of the transition of cations from the steel to the oxide coating is faster than the transport of these ions to the electrolyte solution. Anodic polarization accelerates the oxidation reactions. The oxide layer then grows faster and its thickness increases. Nevertheless, more metal cations pass into the solution. According to Barsoukov[10] and Song[11], the increase in the current density value during anodic polarization reflects the buildup of iron oxides in the passive layer. At some point, the first peak appears, followed by a second, lower one. The first one certainly comes from the oxidation of Cr(III) → Cr(VI), while the second most likely indicates the oxidation of nickel Ni(II) → Ni(III) or manganese Mn(II) → Mn(III). The existence of the electrochemical reactions mentioned above and the build-up of iron oxides is confirmed in the research work by Barsoukov and Song.[10,11] In our previous articles, we assigned a second peak to the iron ion oxidation reaction, which in our present belief is a mistake.[13,14] The results of the XPS tests confirm the above hypotheses. It should be noted that the amount of chromium in the sample decreases after polarization (Table 4). Additionally, the ratio of the intensity of the chromium peaks in the oxide compound to the metallic chromium also decreases. Other important observations are the complete disappearance of the peak indicating the presence of nickel, an increase in the intensity ratio of the iron peaks in the oxide to metallic iron, and the appearance of Mn^{3+} cations. The disappearance of the first and second peak after 80 hours of floating reflects the decrease in the chromium content in the oxide layer and the disappearance of the nickel or the appearance of manganese ions. It should be mentioned that the XPS tests were performed only for CP steel. There is no need to repeat these tests for variably polarized (VP) steel because in both cases the degradation mechanism is the same. The only difference is that, in the case of the VP sample, surface destruction is postponed.

The traces of manganese with the simultaneous absence of nickel are extremely interesting. Song in his important and still up-to-date research work on transpassivation[11] indicates that the peak of secondary passivation is associated with the formation of an inert, electrochemically nonreactive compound, such as $NiFe_2O_4$ and $NiCr_2O_4$ (spinel compounds). Hence, the current density is decreasing. A certain amount of Cr^{3+} and Ni^{2+} ions become trapped

in the crystal lattice of the oxide layer. The results of 316 L stainless steel in this manuscript indicate complete and partial removal of nickel and chromium, respectively, from the oxide layer after deep and long-term surface polarization. Instead, manganese appears. Therefore, in light of the new evidence, it should be considered that chromium is trapped in the form of chromite ($FeCr_2O_4$), while nickel is completely released away from the subsurface layer as a result of the reaction of oxidation to Ni^{3+} ions, which pass into solution. Chromite, as well as the $NiFe_2O_4$ and $NiCr_2O_4$ compounds indicated by Song, is spinel, it is also inert, and it should exhibit the properties of an insulator in the oxide layer.[30] Furthermore, the thermodynamics of a chemical reaction presupposes the possibility of the formation of this compound.[31] Therefore, the presence of chromite is confirmed by XPS analysis. The spectrum obtained shows the compounds containing Cr^{3+} cations, that is, Cr_2O_3 and/or $FeCr_2O_4$. In the case of iron, the charge of the cations is not specified, therefore, they are probably both Fe^{2+} (chromite) and Fe^{3+} (Fe_2O_3). The presence of chromite was also indirectly indicated in our previous article.[32] Raman spectroscopy analysis showed a distinct peak around the wavenumber of 685 cm^{-1}, which can be attributed to both magnetite (Fe_3O_4) and $FeCr_2O_4$. As mentioned already, the results of the XPS tests indicate the presence of manganese in oxide form, containing Mn^{3+}. The first hypothesis, presented above, assumes the occurrence of a nickel oxidation reaction at the potential characteristic for the second peak. The second hypothesis, not less important, defines the second peak as the manganese oxidation reaction to Mn_2O_3 and/or $FeMn_2O_4$, while the latter compound does not exhibit the insulator properties, only half-metal.[33] It should be noted that in the case of distorted oxide structures, the compound formulas expressed here are a kind of simplification. It should be assumed that the coefficients at the atoms of the elements may differ from the stoichiometric values.

References

1. Hirschorn, B.; Orazem, M. E.; Tribollet, B.; Vivier, V.; Frateur, I.; Musiani, M. Determination of effective capacitance and film thickness from constant–phase-element parameters. *Electrochim. Acta* **2010**, 55, 6218–6227. DOI: 10.1016/j.electacta.2009.10.065
2. Béguin, F.; Frąckowiak, E. *Supercapacitors*; Wiley–VCH Verlag GmbH & Co. KGaA, 2013.
3. Wojciechowski, J.; Kolanowski, Ł.; Graś, M.; Szubert, K.; Bund, A.; Fic, K.; Lota, G.; Anti-corrosive siloxane coatings for improved long–term performance of supercapacitors with an aqueous electrolyte. *Electrochim. Acta* **2021**, 372, 137840. DOI: 10.1016/j.electacta.2021.137840
4. Szubert, K.; Wojciechowski, J.; Majchrzycki, Ł.; Jurczak, W.; Lota, G.; Maciejewski, H. The rapeseed oil based organofunctional silane for stainless steel protective coatings. *Materials* **2020**, 13, 2212. DOI: 10.3390/ma13102212
5. Wojciechowski, J.; Baraniak, M.; Lota, G. Highly anti–corrosive treatment of low–carbon steel. *Ceram. Int.* **2021**, 47, 24770–24780. DOI: 10.1016/j.ceramint.2021.05.200
6. Diard, J. P.; Montella, C. Diffusion–trapping impedance under restricted linear diffusion conditions. *J. Electroanal. Chem.* **2003**, 557, 19–36. DOI: 10.1016/S0022-0728(03)00346-2

7. Bisquert, J.; Garcia-Belmonte, G.; Bueno, P.; Longo, E.; Bulhoes, L. O. S. Impedance of constant phase element (CPE)-blocked diffusion in film electrodes. *J. Electroanal. Chem.* **1998**, 452, 229–234. DOI: 10.1016/S0022-0728(98)00115-6
8. Remita, E.; Boughrara, D.; Tribollet, B.; Vivier, V.; Sutter, E.; Ropital, F.; Kittel, J. Diffusion impedance in a thin-layer cell: experimental and theoretical study on a large-disk electrode. *J. Phys. Chem. C* **2008**, 112, 4626–4634. DOI: 10.1021/jp710407a
9. Lasia, A. *Electrochemical Impedance Spectroscopy and its Applications*; Springer, 2015.
10. Barsoukov, E.; Macdonald, J. R. *Impedance Spectroscopy: Theory, Experiment, and Applications,* 2nd Edition; Wiley, 2005.
11. Song, G. Transpassivation of Fe–Cr–Ni stainless steels. *Corros. Sci.* **2005**, 47, 1953–1987. DOI: 10.1016/j.corsci.2004.09.007
12. Przygocki, P.; Abbas, Q.; Babuchowska, P.; Beguin, F. Confinement of iodides in carbon porosity to prevent from positive electrode oxidation in high voltage aqueous hybrid electrochemical capacitors. *Carbon* **2017**, 125, 391–400. DOI: 10.1016/j.carbon.2017.09.060
13. Znaniecki, S.; Szwabińska, K.; Wojciechowski, J.; Skrzypczak, A.; Lota, G. Ionic liquid modified electrochemical capacitor with long–term performance. *ChemElectroChem* **2021**, 8, 3685–3694. DOI: 10.1002/celc.202100573
14. Znaniecki, S.; Szwabińska, K.; Wojciechowski, J.; Skrzypczak, A.; Baraniak, M.; Lota, G. Capacitor lifetime prolonged by addition of organic ammonium salt with cyclohexyl substituent and 2,5–dihydroxybenzenesulfonic anion. *Electrochem. Commun.* **2022**, 140, 107326. DOI: 10.1016/j.elecom.2022.107326
15. http://www.madehow.com/Volume-1/Stainless-Steel.html (accessed 2023-09-08)
16. https://fractory.com/stainless-steel-finishes-en-astm/ (accessed 2023-09-08)
17. https://www.gasparini.com/en/blog/mechanical-finishes-of-stainless-steel/ (accessed 2023-09-08)
18. http://www.dsstainlesssteel.com/stainless-steel-finishes/ (accessed 2023-09-08)
19. Fenili, C. P.; da Rocha, M. R.; Al-Rubaie, K. S.; Arnt, Â. B. C.; Angioleto, E.; Bernardin, A. M. Effect of sensitization on tribological behavior of AISI 304 austenitic stainless steel. *Int. J. Mater. Res.* **2018**, 109, 234–240. DOI: 10.3139/146.111594
20. Okazaki, Y. Characterization of oxide film of implantable metals by electrochemical impedance spectroscopy. *Materials* **2019**, 12, 3466. DOI: 10.3390/ma12213466
21. Gardin, E.; Zanna, S.; Seyeux, A.; Allion-Maurer, A.; Marcus, P. Comparative study of the surface oxide films on lean duplex and corresponding single phase stainless steels by XPS and ToF-SIMS. *Corros. Sci.* **2018**, 143, 403–413. DOI: 10.1016/j.corsci.2018.08.009
22. Tardio, S.; Abel, M.–L.; Carr, R. H.; Castle, J. E.; Watts, J. F. Comparative study of the native oxide on 316L stainless steel by XPS and ToF-SIMS. *J. Vac. Sci. Technol. A* **2015**, 33, 05E122. DOI: 10.1116/1.4927319
23. Olesen, B. H.; Avci, R.; Lewandowski, Z. Manganese dioxide as a potential cathodic reactant in corrosion of stainless steels. *Corros. Sci.* **2000**, 42, 211–227. DOI: 10.1016/S0010-938X(99)00071-2
24. Perez, N. *Electrochemistry and Corrosion Science*; Kluwer, 2004.
25. McCafferty, E. *Introduction to Corrosion Science*; Springer-Verlag, 2010.
26. Groysman, A. *Corrosion for everybody*; Springer, 2010.
27. Revie R. W.; Uhlig, H. H. *Corrosion and Corrosion Control: An Introduction to Corrosion Science and Engineering*; John Wiley & Sons, Inc., 2008.
28. Revie, R. W. *Uhlig's Corrosion Handbook*; John Wiley & Sons, Inc., 2011.
29. Olsson, C.-O. A.; Landolt, D. Passive films on stainless steels – chemistry, structure and growth. *Electrochim. Acta* **2003**, 48, 1093–1104. DOI: 10.1016/S0013-4686(02)00841-1
30. Chambers, S. A.; Droubay, T. C.; Kaspar, T. C.; Nayyar, I. H.; McBriarty, M. E.; Heald, S. M.; Keavney, D. J.; Bowden, M. E.; Sushko, P. V. Electronic and optical properties of a semiconducting spinel (Fe_2CrO_4). *Adv. Funct. Mater.* **2017**, 27, 1605040. DOI: 10.1002/adfm.201605040
31. Qi, T.–G.; Liu, N.; Li, X.–B.; Peng, Z.–H.; Liu, G.–H.; Zhou, Q.–S. Thermodynamics of chromite ore oxidative roasting process. *J. Cent. South Univ. Technol.* **2011**, 18, 83−88. DOI: 10.1007/s11771−011−0663−0
32. Wojciechowski, J.; Kolanowski, Ł.; Bund, A.; Lota, G. The influence of current collector corrosion on the performance of electrochemical capacitors. *J. Power Sources* **2017**, 368, 18–29. DOI: 10.1016/j.jpowsour.2017.09.069
33. Santos-Carballal, D.; Roldan, A.; Grau-Crespo, R.; de Leeuw, N. H. First–principles study of the inversion thermodynamics and electronic structure of FeM_2X_4 (thio)spinels (M = Cr, Mn, Co, Ni; X = O, S). *Phys. Rev. B* **2015**, 91, 195106. DOI: 10.1103/PhysRevB.91.195106
